# The impact of TMS and PNS frequencies on MEP potentiation in PAS with high-frequency peripheral component

**DOI:** 10.1371/journal.pone.0233999

**Published:** 2020-05-29

**Authors:** Magdolna Mezes, Roope Havu, Aleksandra Tolmacheva, Pantelis Lioumis, Jyrki P. Mäkelä, Anastasia Shulga

**Affiliations:** 1 Department of Neurology, Christian Doppler Medical Centre, Paracelsus Medical University and Centre for Cognitive Neuroscience, Salzburg, Austria; 2 BioMag Laboratory, HUS Medical Imaging Center, University of Helsinki and Helsinki University Hospital, Helsinki, Finland; 3 Department of Neuroscience and Biomedical Engineering, Aalto University School of Science, Helsinki, Finland; 4 Clinical Neurosciences, Neurology, University of Helsinki and Helsinki University Hospital, Helsinki, Finland; Universita degli Studi di Catania, ITALY

## Abstract

Paired associative stimulation (PAS) combines transcranial magnetic stimulation (TMS) and peripheral nerve stimulation (PNS) to induce plastic changes in the corticospinal tract. PAS employing single 0.2-Hz TMS pulses synchronized with the first pulse of 50–100 Hz PNS trains potentiates motor-evoked potentials (MEPs) in a stable manner in healthy participants and enhances voluntary motor output in spinal cord injury (SCI) patients. We further investigated the impact of settings of this PAS variant on MEP potentiation in healthy subjects. In experiment 1, we compared 0.2-Hz *vs* 0.4-Hz PAS. In experiment 2, PNS frequencies of 100 Hz, 200 Hz, and 400 Hz were compared. In experiment 3, we added a second TMS pulse. When compared with 0.4-Hz PAS, 0.2-Hz PAS was significantly more effective after 30 minutes (p = 0.05) and 60 minutes (p = 0.014). MEP potentiation by PAS with 100-Hz and 200-Hz PNS did not differ. PAS with 400-Hz PNS was less effective than 100-Hz (p = 0.023) and 200-Hz (p = 0.013) PNS. Adding an extra TMS pulse rendered PAS strongly inhibitory. These negative findings demonstrate that the 0.2-Hz PAS with 100-Hz PNS previously used in clinical studies is optimal and the modifications employed here do not enhance its efficacy.

## Introduction

Several studies have recently demonstrated the potential therapeutic applications of non-invasive brain stimulation, including transcranial magnetic stimulation (TMS) [1, 2]. Non-invasive depolarization of neuronal membranes by TMS initiates action potentials and enables targeting and modulation of activity of cortical neuronal ensembles. Activation or suppression of neuronal activity provides therapeutic opportunities for numerous neurological conditions [1]. Paired associative stimulation (PAS) combines TMS of the primary motor cortex (M1) with electrical peripheral nerve stimulation (PNS) of the contralateral extremities. The potential of PAS as a therapeutic tool has been studied in stroke [3], neurodegenerative disorders [4] and spinal cord injury [5] patients, among others [6].

Long-term-potentiation (LTP) is a cellular mechanism that induces long-lasting increase of synaptic efficacy and neuroplasticity [7]. LTP occurs as a consequence of simultaneous activity of pre-and postsynaptic cells [8]. *N*-methyl-d-aspartate (NMDA) channel-dependent LTP provides an attractive cellular model of learning and memory and may play an essential role in developing functional neural networks [9, 10]. The aim of PAS is to create the conditions that can contribute to induction of LTP *in vivo*. If the timing between the two stimuli (inter-stimulus interval, ISI) is appropriate, PNS signals that ascend via sensory volley to M1 coincide with the TMS-induced neural impulses from M1. This coincidence can transiently increase the corticospinal excitability. [11, 12] In spinal PAS, antidromic and orthodromic signals are timed to occur simultaneously at the spinal cord level. This repeated pairing of signals is thought to induce an LTP-like effect at the corticospinal-motoneuronal synapses [13–15].

TMS and PAS protocols can engage corticospinal plasticity and are under investigation as a tool to enhance motor function after spinal cord injury (SCI), which is rarely complete [5]. We have shown in several case reports and series that PAS with a high-frequency peripheral component (0.2-Hz TMS paired with 100-Hz PNS) enhances motor output of paretic or paralytic muscles in patients with chronic incomplete SCI [16–19]. At the moment this is the only PAS protocol variant that has produced clinically meaningful and long-lasting improvements in patients with SCI. Previous studies have applied PAS to spinal cord injury patients as single sessions only [13–15]. In stroke patients, conventional PAS applied for 4 weeks improved some neurophysiological and functional measures [20].

The potential for PAS to increase or decrease excitability strongly depends on the interval between TMS and PNS pulses [12][21]. Therefore, precise timing between the two stimuli is crucial. Conventional PAS (single-pulse TMS combined with single pulse or 10-Hz PNS) protocols employ either fixed ISI (across participants) or individually determined ISIs [21][6]. The variable outcomes of conventional PAS reflect its dependence on multiple technical and individual factors such as time of day, pre-PAS activity, and subject characteristics [6]. Patients with SCI may have longer neuronal conduction times in both orthodromic and antidromic pathways, whose conductivity may also change during the rehabilitation and time since injury. Therefore, finding the precise ISI and most optimal parameters of PAS protocol can be particularly challenging. Employing a PAS protocol at 0.2-Hz with high-intensity TMS (100% of the stimulator output) and high-frequency peripheral stimulation leads reliably to robust motor-evoked potential (MEP) potentiation at a wide range of ISIs, plausibly due to increase in collision events between TMS- and PNS-induced neuronal impulse volleys [16, 17] PAS with a 100-Hz PNS appears to be the most effective [16][22, 23].

Further development of PAS variants with a high-frequency peripheral component is of clinical interest. We searched for an increase of the excitatory effect and decrease of the time required for PAS by modifying the previously employed”standard” protocol (0.2-Hz single pulse TMS, 240 stimuli in 20 minutes on the M1, paired with 100-Hz PNS to the right tibial nerve). We wanted to achieve the same or higher MEP potentiation in a more time-efficient manner and compared the potentiation induced with several modified versions with the effects of the standard protocol.

The rationale of this study was to test whether increasing the frequency of either PAS or the PNS component of PAS or doubling the amount of TMS pulses would enhance the efficacy, feasibility, or both of the protocol that we have used in clinical studies. The aim of all experiments was either to show the superiority of new PAS modifications or to conclude that the current version of PAS with a high-frequency peripheral component (currently under investigation for clinical use) is currently the most optimal choice for PAS. Increasing the PAS frequency would reduce the time of the PAS protocol and render it more feasible for clinical use and may also increase its efficacy. Since PAS aims at the coincidence of ascending and descending volleys at the spinal cord level, we also hypothesized that increasing the frequency of PNS component, or increasing the number of TMS pulses, could further increase PAS efficacy by enhancing the number of coinciding volleys. A single high-intensity TMS pulse produces several descending volleys, a D-wave, and four I-waves at a frequency of approximately 500–660 Hz [24]. We have previously shown that increasing the frequency of the PNS component from 50 Hz to 100 Hz enhances the efficacy of PAS.

## Participants and methods

### Participants

The study was approved by the Ethics Committee of Helsinki University Hospital (HUS/1280/2016). Twenty healthy participants without contraindication for TMS were recruited. Some subjects participated in more than one experiment. Each subject signed an informed consent form before participation. All experiments were performed according to relevant guidelines and regulations [25]. All subjects were right-handed. The average (±SD) height and weight of the subjects was 170±9 cm, 69±11 kg; 174±4 cm, 66±9 kg and 167±9 cm, 72±14 kg for Experiment 1, 2, and 3, respectively. The education level of the subjects for Experiments 1, 2, and 3 was as follows: undergraduates (33, 30, 40%), MSc or MD (44, 30, 40%), PhD (22, 40, 20%).

### Transcranial magnetic stimulation

TMS pulses were generated with an eXimia magnetic stimulator employing a figure-of-eight coil (Nexstim Ltd., Helsinki, Finland). We applied MRI-guided TMS navigation (Navigated Brain Stimulation 4.3 [NBS 4.3], Nexstim Ltd., Helsinki, Finland) based on 3D models of the individual 3T T1 MRI images. In a prospective series of patients, comparison of the preoperative and intraoperative localization of hand motor cortex yielded distances of 4–14 mm between nTMS and direct cortical stimulation. [26–29] Navigation guarantees the accurate localisation of M1 and the precise repetition of the same cortical location with exactly the same coil positioning and orientation, securing the same induced electric field throughout the whole session and between different experimental sessions. The TMS coil was positioned over the left primary M1 to activate the “hotspot” of the right abductor hallucis muscle. During the mapping, we systematically recorded MEPs from the whole motor representation area of the distal lower limb. We defined the hotspot as a site where TMS pulses provided the maximal and most consistent MEPs from the right abductor hallucis muscle and induced a plantar flexion. MEPs were recorded and analysed with an EMG device integrated in the eXimia stimulator. The resting motor threshold (RMT) of the contralateral abductor hallucis muscle was defined as the minimum TMS intensity required to evoke a MEP of >50 *μ*V in at least 5 of 10 trials over the “hotspot”. During PAS, an intensity of 100% of maximum stimulator output (MSO) was used to mimic the conditions of studies where this protocol was applied to SCI patients [16–19]. The MEP measurements were performed with 120% of individual RMT. Individual RMTs of the participants are presented in Table 1. RMTs in the three experiments did not differ significantly (p = 0.114 by Kruskal-Wallis test). MEP latency was calculated from an average of 10 MEPs elicited at an interval of 3.3 s at 120% RMT. The average of MEP latencies was used to calculate the ISI (F-MEP_average_) [30] between the TMS and PNS pulses.

**Table 1:** Individual resting motor thresholds (RMTs), peripheral nerve stimulation (PNS) intensities, and pre-PAS motor-evoked potential (MEP) amplitudes in three experiments. RMTs and PNS intensities were defined once prior to all measurements of the experiment. Pre-PAS MEP amplitudes are the average values of all pre-PAS measurements included in one experiment for each subject.

Subjects	Experiment 1			Subjects	Experiment 2			Subjects	Experiment 3		
	RMT	PNS	pre-PAS		RMT	PNS	pre-PAS		RMT	PNS	pre-PAS
		intensity	MEP			intensity	MEP			intensity	MEP
			amplitude				amplitude				amplitude
	% SO	mA	μV		% SO	mA	μV		% SO	mA	μV
1	88	13	289	1	76	11	154	1	88	13	180
2	49	6	316	2	71	5	553	2	81	12	444
3	36	5,4	595	3	76	14	752	3	96	6	222
4	90	8,5	514	4	87	6	89	4	91	7,5	314
5	98	12	859	5	52	17	495	5	90	8,5	884
6	96	6	127	6	65	20	337				
7	91	7,5	545	7	100	15	44				
8	70	8,5	258	8	52	9,7	405				
9	81	12	600	9	73	15	449				
				10	51	10	880				
**median**	88	8,5	514		72	12,5	427		90	8,5	314
**mean**	78	8,8	456		70	12,3	416		89	9,4	409
**SD**	22	2,9	226		16	4,8	274		5	3,0	284

### Electrical peripheral nerve stimulation

PNS was delivered using a Dantec Keypoint electroneuromyography device (Natus Medical Inc., Pleasanton, CA, USA). The tibial nerve was stimulated with two surface electrodes (Neuroline 720, AMBU A/S, Ballerup, Denmark) positioned at the medial side of the ankle, between the medial malleolus and the Achilles tendon. Before stimulation, EMLA Cream (lidocaine 2.5% and prilocaine 2.5%) was applied locally at the stimulation site for 16 participants to reduce the sensations produced by PNS. Although all participants were offered EMLA, only 16 chose to use it. EMLA penetrates 3–5 mm into the skin [31] and thus does not affect the conductivity of the tibial nerve. The same surface electrodes were employed for the F-response recording. The recording electrode was placed over the belly of abductor hallucis muscle and the reference electrode on the medial side of the hallux. Ten F-responses were recorded with a single 0.2-ms stimulation at supramaximal intensity. From these responses, the one with the shortest F-latency was selected and used for ISI calculation (F-MEP_average_). Square wave pulses of 1 ms were applied to identify the individual minimum intensity evoking the F-response. This intensity was used for PNS in PAS. PNS intensities of each participant are presented in Table 1. PNS intensities in three experiments did not differ significantly (p = 0.196 by Kruskal-Wallis test). Trains of six 1-ms square wave pulses were delivered at 100–400 Hz.

### Paired associative stimulation

PNS and TMS were triggered by Presentation^®^ software (Neurobehavioral Systems Inc., Albany, NY, USA) to ensure their precise timing. Each TMS pulse was paired with a PNS train. ISIs between the TMS and the first pulse of the PNS train were calculated with the formula (F-MEP_average_) as described previously [30]. To mimic the conditions of studies where this protocol was applied to SCI patients [16–19], all participants were asked to imagine plantar flexion of the right foot during the PAS session.

### Experimental design

**Fig 1:**
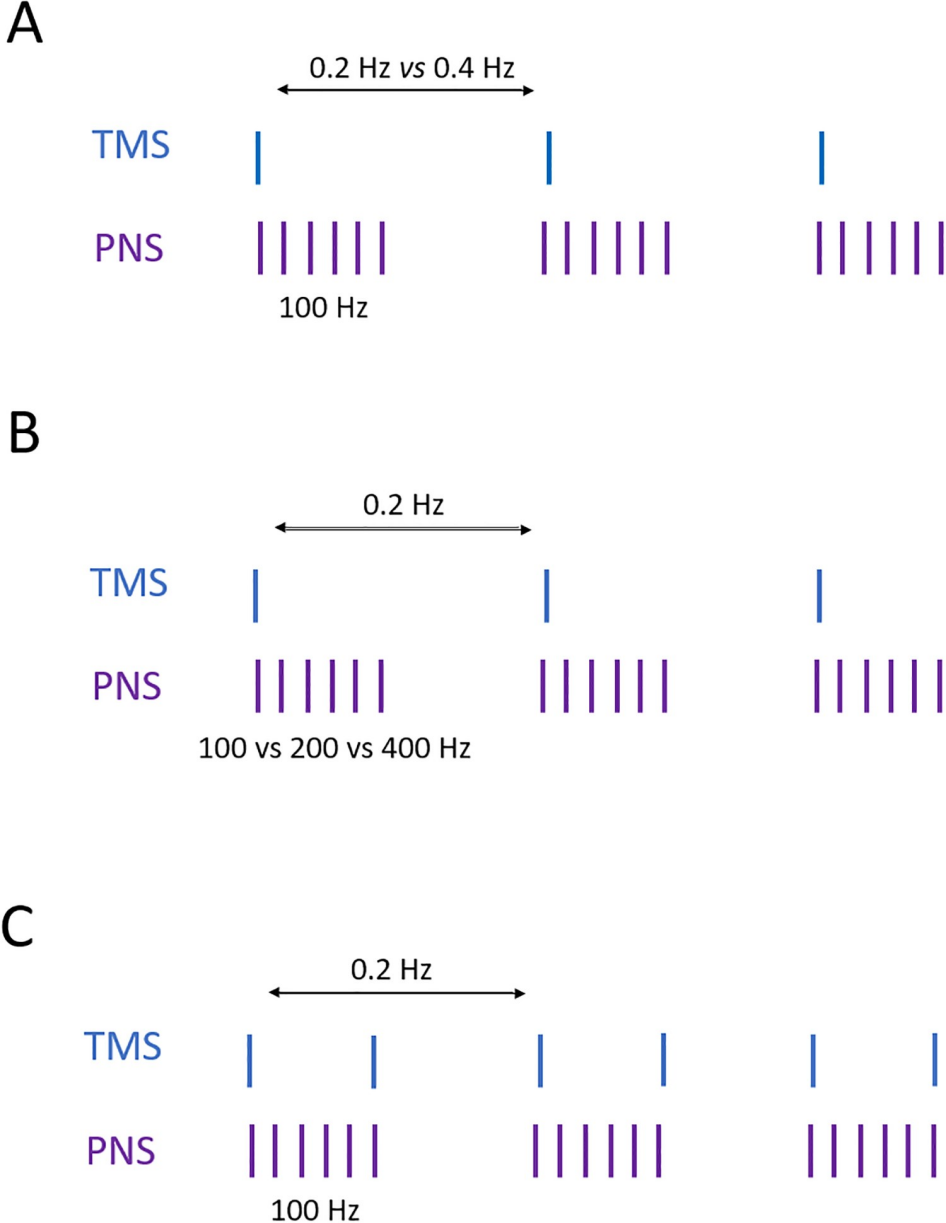
Schematic representation of TMS and PNS parameters tested in A) Experiment 1; B) Experiment 2, and C) Experiment 3.

Experiment 1 (Fig 1A) compared the 20-min 0.2-Hz TMS protocol with the 10-min 0.4-Hz protocol on MEP potentiation at 0, 30, and 60 min after PAS. A total of 240 single TMS pulses were delivered in both protocols (once every 5 s or 2.5 s, respectively). Nine healthy participants were recruited (6 females, age range 22–42 years, mean age 32 years). Each participant had a PAS session on two different days separated by at least 7 days. The two protocols were applied in a random order.

Experiment 2 (Fig 1B) compared 0.2-Hz PAS with 100-Hz, 200-Hz, and 400-Hz PNS components on MEP potentiation at 0, 30, and 60 min after PAS. Ten healthy participants were recruited (5 females, age range 22–46 years, mean age 37 years). Each participant had a PAS session on three different days separated by at least 7 days. The three protocols were applied in a random order.

In Experiment 3 (Fig 1C), we added a second TMS pulse 50 ms after the first one, pairing the first and the second TMS pulses with the first and sixth PNS pulses, respectively, at the level of the spinal cord. Both pulses were given at 96% of MSO due to safety limitations of the TMS device. We examined whether the increase in the number of orthodromic volleys could further enhance MEP potentiation. Five healthy participants (three females, age range 30–39, mean age 34) were enrolled. Each participant underwent one session of PAS.

In all experiments, the MEP amplitude changes were assessed from an average of 30 MEPs elicited with TMS delivered to the hotspot of right abductor hallucis muscle once every 3.3 s at 120% of RMT. Assessments were conducted immediately prior to the PAS session, immediately post-session (0 min), 30 min post-session, and 60 min post-session. MEP potentiation was calculated as a percent ratio of an average of post-PAS normalized to pre-PAS MEP amplitudes. EMG was recorded continuously and analysed 200 ms prior to MEPs to detect muscle preactivation. MEPs with preactivation were excluded from the analysis.

### Statistical analysis

Statistical analysis was performed using SPSS 25.0. An average of 30 MEPs was calculated at each timepoint post-PAS and compared with the averaged value of amplitudes from 30 MEPs measured before the PAS session; percent ratios post-PAS/pre-PAS were defined. Data were assessed with Wilcoxon signed-rank test and with Friedman test for multiple comparisons.

## Results

**Fig 2:**
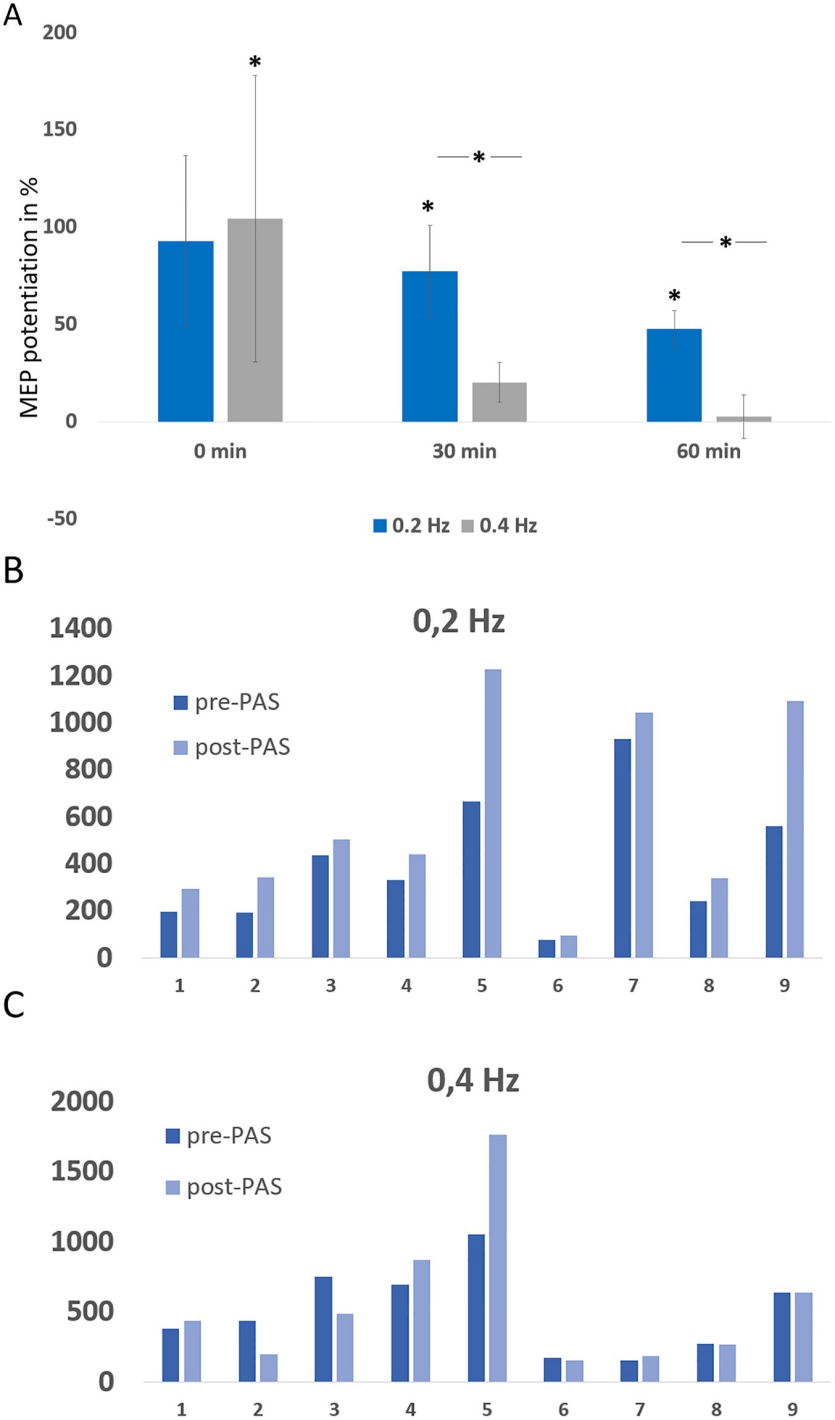
Experiment 1. A, MEP potentiation (post-PAS normalized to pre-PAS minus 100%) induced by PAS delivered at 0.2 Hz and 0.4 Hz. At 30 min and 60 min, the 0.4-Hz protocol did not enhance MEPs, whereas the 0.2-Hz protocol generated MEP potentiation. The 0.2-Hz protocol induced a significantly stronger MEP potentiation than the 0.4-Hz protocol at 30 and 60 min. B-C) MEP values at pre-PAS and 60 min post-PAS induced by 0.2 Hz (B) and 0.4 Hz (C) in individual participants in Experiment 1.

Experiment 1 (Fig 1A) compared the MEP potentiation induced by 0.4-Hz and 0.2-Hz PAS (Fig 2). At 0 min post-PAS, the 0.4-Hz protocol induced a significant MEP potentiation (p = 0.038, 204±73%). A trend towards MEP potentiation with the 0.2-Hz protocol was observed (p = 0.66, 193±43%). At 30 min and 60 min, the 0.4-Hz protocol did not enhance MEPs (30 min, p = 0.066; 60 min, p = 0.77) whereas the 0.2-Hz protocol generated a significant long-term MEP potentiation (30 min, p = 0.008, 177±24%; 60 min, p = 0.008, 147±10%), consistent with our previous results [23]. The 0.2-Hz protocol induced a significantly stronger MEP potentiation than the 0.4-Hz protocol at 30 min (p = 0.05) and 60 min (p = 0.011). At 0 min (p = 0.3), no significant difference was found (Fig 2A). Individual values for each subject at pre-PAS and 60 min post-PAS are shown in Fig 2B and 2C.

**Fig 3:**
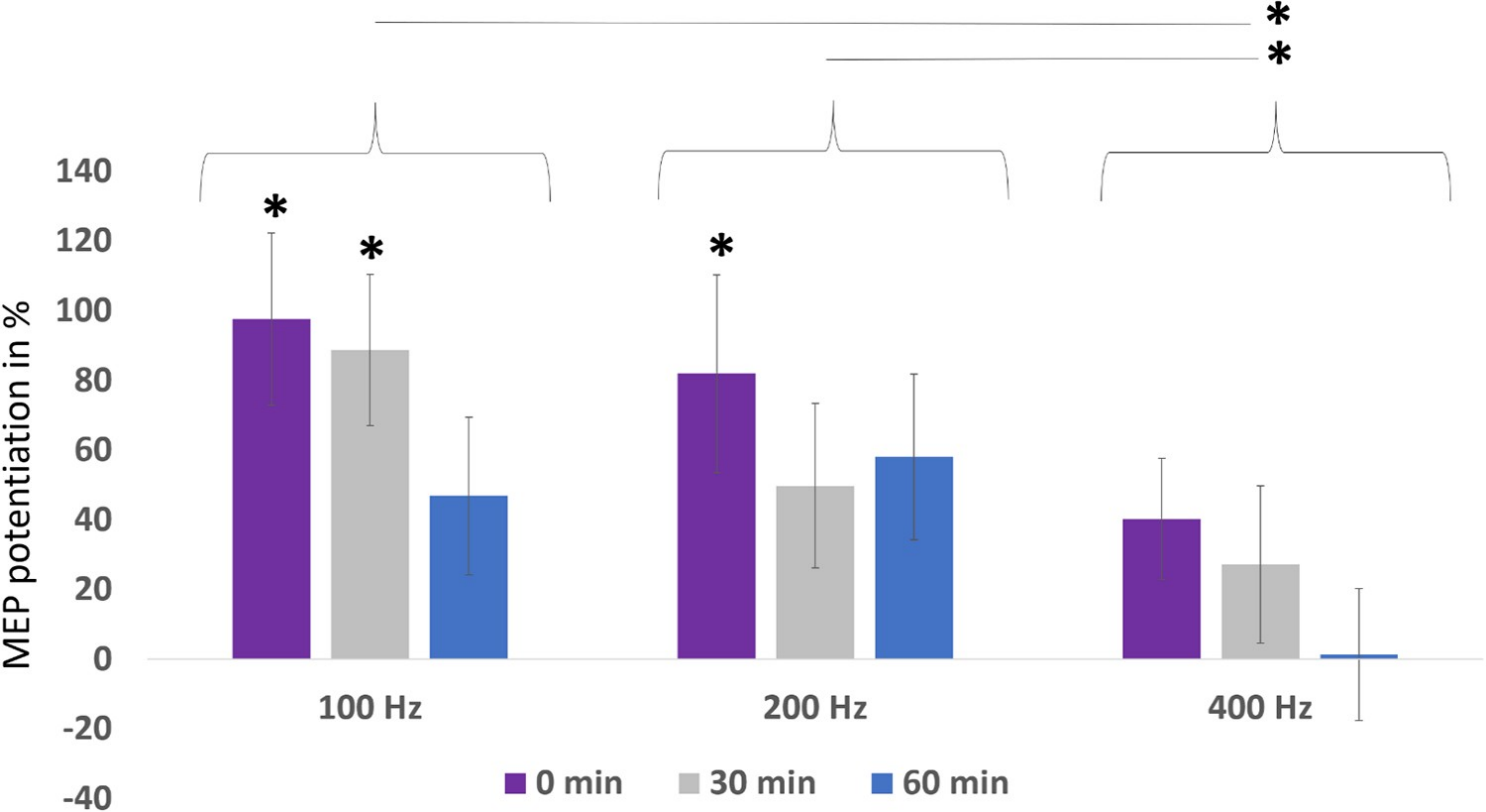
Experiment 2. MEP potentiation (post-PAS normalized to pre-PAS minus 100%) induced by PAS with 100-Hz, 200-Hz, and 400-Hz PNS. The significant MEP potentiation was induced by the 100-Hz protocol at 0 and 30 min, and by the 200-Hz protocol at 0 min. The 400-Hz protocol did not induce significant MEP potentiation. PAS with PNS of 400 Hz induced significantly weaker MEP potentiation than PAS with 100 Hz and 200 Hz PNS.

In Experiment 2 (Fig 1B), we compared MEP potentiation up to 60 min after PAS with PNS of 100 Hz, 200 Hz, and 400 Hz. The 100-Hz protocol induced a significant MEP potentiation at 0 min (p = 0.005; 198±25%) and 30 min (p = 0.009; 189±28%); the 200-Hz protocol induced a significant MEP potentiation only at 0 min (p = 0.022; 182±22%); and the 400-Hz protocol did not induce significant MEP potentiation at any time point (Fig 3). A Friedman test including all timepoints revealed a significant difference between the groups (p = 0.048). The protocol with 400-Hz PNS induced significantly weaker potentiation at all time points than 100 Hz (p = 0.023) and 200 Hz (p = 0.013). The 100-Hz and 200-Hz protocols did not differ (p = 0.6) (Fig 3).

**Fig 4:**
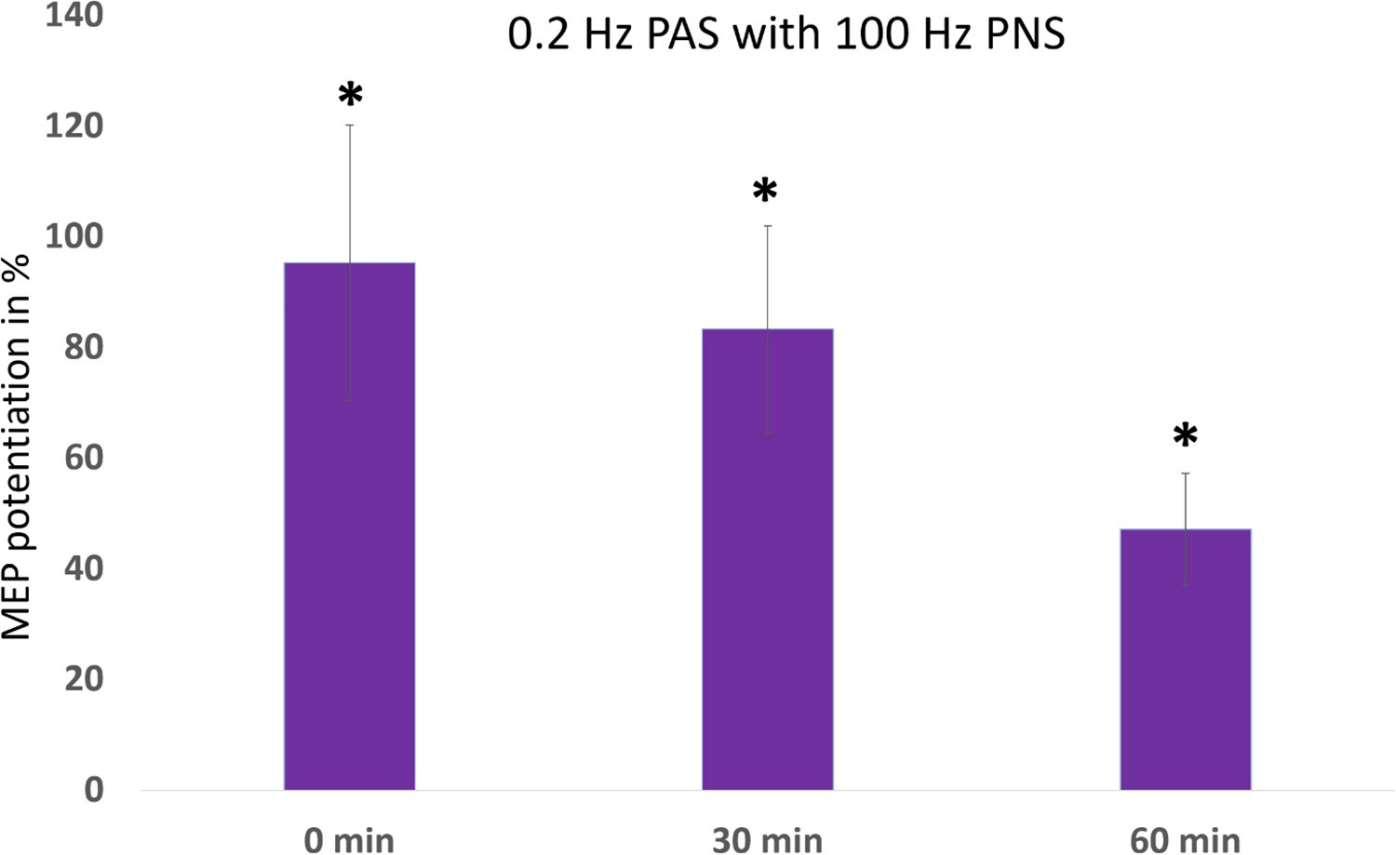
MEP potentiation (post-PAS normalized to pre-PAS minus 100%) induced by 0.2-Hz protocol with 100-Hz PNS, pooled data from Experiments 1 and 2, n = 19. The protocol induced significant MEP potentiation at all time points.

Joint analysis of 0.2-Hz PAS with 100-Hz PNS (the “standard” protocol) from experiments 1 and 2 (Fig 4, n = 19 measurements) showed a significant MEP potentiation at all timepoints. According to the Friedman test, MEP potentiation was significantly different between timepoints (p < 0.0001). Significant differences were found with post-hoc analysis by Wilcoxon signed-rank tests between pre-PAS and all other timepoints (0 min, p = 0.001, 195±25%; 30 min, p < 0.0001, 183±19%; 60 min, p = 0.002, 147±10%), consistent with our previous results [23].

Experiment 3 examined whether the MEP potentiation of the 0.2-Hz protocol can be further increased by adding an another TMS pulse. We found a clear inhibitory effect when this modified PAS was employed (0 min, -64±14%, p = 0.043; 30 min, -66±20%, p = 0.23; 60 min, -52±12%, p = 0.043).

## Discussion

The aim of this study was to investigate whether any of the PAS variants studied here is superior to the previously applied, clinically beneficial protocol [16–19]. Negative results were obtained, showing that the clinical protocol is the most efficient; neither non-superiority nor inferiority of the new PAS protocols presented here can be concluded from the obtained data. In experiment 1, significantly weaker MEPs at 30 min and 60 min were detected after applying the shorter than clinical PAS protocol. In experiment 2, a significant difference was detected between all PAS protocols. Specifically, the difference was detected between the 100-Hz and 400-Hz PAS protocols; the 400-Hz protocol elicited significantly weaker MEPs than the 100-Hz protocol. In experiment 3, a clear significant MEP inhibition by adding a second TMS pulse was found. Therefore, the protocol version of PAS with a high-frequency peripheral component, currently applied in clinical studies, is currently the most efficient protocol.

The temporal relationship between the activations of pre- and postsynaptic neurons appear to dictate the extent and polarity of plastic changes, known as spike timing-dependent plasticity (STDP) [32]. LTP also may depend on firing rate [7] or combination of firing rate, spike timing, and co-cooperativity among the inputs [33]. The situation *in vivo* is substantially more complex than in cellular models, as complex patterns of neural activity of the motor cortex are involved. This leads to variable outcomes in conventional PAS protocols, thus highlighting strong dependence on external conditions [6]. In designing PAS protocols that are feasible for neurological rehabilitation, clinical challenges must be considered. Changes in signal conduction time in both orthodromic and antidromic pathways [34] and measurement inaccuracies in MEPs due to muscle spasticity are expected in patients with SCI. Considering all these factors, optimising the PAS protocol for SCI patients is very challenging. We have previously compared the 0.2-Hz PAS protocol with 50-Hz PNS that was employed in studies involving patients with incomplete chronic SCI [16–17] with a protocol involving 100-Hz PNS that was shown to be more effective [23]. Here, we wanted to investigate whether similar efficacy can be obtained in a shorter time or improved by further modification of PNS.

In Experiment 1 we halved the duration of the PAS session by increasing the PAS frequency to 0.4 Hz. This modification induced significantly weaker MEPs at 30 and 60 min after PAS than the 0.2-Hz protocol. The results of Experiment 1 might be due to the impact of stimulation duration, its frequency, or both. Some studies did apply facilitating PAS with a duration of 10 min or shorter to induce LTP at the cortical [35] and spinal level [13]. However, the efficacy of these protocols was not examined at 30 and 60 min after the PAS. In in vitro experiments, the high-frequency stimulation induces an activity-dependent release of brain-derived neurotrophic factor (BDNF), known to play a crucial role in the LTP induction [36–38]. In a study where vagus nerve stimulation (VNS) was paired with an auditory stimulus for inducing recovery-promoting plasticity in the auditory cortex [39], shortening the interval between VNS tone-pairing events also reduced the plastic response and led to loss of the therapeutic effect of the stimulation [39]. The authors concluded that longer intervals between VNS tone-pairing events generate more plasticity and better recovery because the structural changes that underlie these improvements require many seconds to minutes to develop [39]. The possible role of activity-dependent plasticity-inducing molecules in the PAS effect might explain why a sufficiently low frequency of PAS is required. A frequency that is too high might deplete relevant components of the neurotrophin release machinery, such as vesicles and calcium stores, not allowing sufficient time for plastic response to occur, and therefore rendering particularly the long-term plasticity less effective. Experiment 2 demonstrated that although 100-Hz PNS is more efficient than 50- and 25-Hz PNS [23], a further increase in frequency of PNS does not provide additional efficacy. Thus, bringing the PNS frequency closer to the frequency of I-waves [24] does not produce stronger potentiation; the exact coincidence of each PNS pulse with each TMS-induced volley does not appear to be the strongest determining factor for MEP potentiation. Rather, the specific pattern of PNS appears to be important, although PNS by itself does not produce MEP potentiation [23]. Consistent with the result of Experiment 1, the highest frequency is not the most efficient. Activity-dependent release from the peripheral motoneuronal pool of neurotrophic factors such as BDNF is known to occur at 50–100 Hz [36–38]. Frequencies higher than 100 Hz might not be as effective due to depletion of relevant components of the neurotrophin release machinery, as mentioned above.

During selection of TMS parameters in Experiment 3, we aimed at a precise pairing of the second TMS pulse with one of the PNS pulses of the PNS train. In addition, we aimed to apply the same or similar stimulation intensity that was used in the 0.2-Hz protocol (100% MSO) to ensure the comparability of the results. The 20-Hz TMS was selected to achieve as high a TMS intensity as feasible. The safety guidelines of our TMS device requires a reduction of intensity as the applied frequency increases. Employing this frequency enabled a maximum intensity at 96% of MSO and a precise pairing also with the sixth stimulus of the PNS train. The inhibitory effect found in Experiment 3 most probably reflects the long interval intra-cortical inhibition (LICI) that occurs by employing paired pulse TMS with ISIs between 50 and 200 ms, generally considered to be mediated by cortical GABAb receptors [40]. The cortical LICI effect most probably induces metaplastic change [41] in the motor pathways, preventing PAS facilitatory effects. It has been suggested previously by pharmacological studies that the GABAb receptor agonist baclofen decreases PAS-induced LTP-like plasticity in the human motor cortex [42]. GABAb inhibitory postsynaptic potentials may explain why we observed a negative impact on the MEP amplitudes in this study. However, the increase in the number of orthodromic volleys by applying several TMS pulses might contribute to a more effective PAS protocol when delivered with other intervals and needs further examination.

A limitation of this study is that no significant difference was detected between the 100-Hz and 200-Hz protocols in Experiment 2. However, it is evident from Fig 3 that the 200-Hz protocol is not superior to the 100-Hz protocol. However, it is not clear if the 200-Hz PNS is significantly inferior to the 100-Hz PNS; a larger sample size is required to answer this question. As our aim was to find protocols superior to PAS with a 100-Hz PNS component, this question is not clinically urgent. Moreover, we investigated only one modification of PAS frequency and two modifications of PNS frequency. Although our clinical test protocol of PAS with high-frequency PNS is the most efficient protocol among the options studied here, it remains open whether this protocol can nevertheless be further optimized. Further studies that reveal the exact mechanisms of action of the protocols and include more protocol variants with modifications of TMS, PNS, and PAS frequencies and intensities are needed.

These results combined with clinical studies [16–19] suggest that 0.2-Hz PAS with 100-Hz PNS can be applied for patients with incomplete SCI to improve their motor function. More studies are needed to optimize the timing and duration of the treatment and patient selection. Current data [16–19] suggest that a longer stimulation time, earlier initiation of treatment, and milder injury may be associated with better outcomes. More research is needed to confirm these hypotheses and to further optimize the applied PAS protocol.

## Conclusions

None of the modified paired-associative protocols that we examined in this study could provide a stronger long-term MEP potentiation than the one we have applied previously [16–19]. Our findings indicate that 0.2-Hz TMS PAS employing 240 single TMS stimuli on M1 paired with 100-Hz PNS is the most effective protocol of PAS employing high-frequency PNS.
